# The regulation and potential roles of m6A modifications in early embryonic development and immune tolerance at the maternal-fetal interface

**DOI:** 10.3389/fimmu.2022.988130

**Published:** 2022-09-26

**Authors:** Hong Liu, Jie Zheng, Aihua Liao

**Affiliations:** ^1^ Department of Reproduction, Maternal and Child Health Hospital of Hubei Province, Affiliated in Tongji Medical College, Huazhong University of Science and Technology, Wuhan, China; ^2^ Institute of Reproductive Health, Center for Reproductive Medicine, Tongji Medical College, Huazhong University of Science and Technology, Wuhan, China

**Keywords:** m6A methylation, embryo development, immune tolerance, maternal-fetal interface, early pregnancy

## Abstract

The immune microenvironment at the maternal-fetal interface was determined by the crosstalk between the trophoblast and maternal-derived cells, which dynamically changed during the whole gestation. Trophoblasts act as innate immune cells and dialogue with maternal-derived cells to ensure early embryonic development, depending on the local immune microenvironment. Therefore, dysfunctions in trophoblasts and maternal decidual cells contribute to pregnancy complications, especially recurrent pregnancy loss in early pregnancy. Since many unknown regulatory factors still affect the complex immune status, exploring new potential aspects that could influence early pregnancy is essential. RNA methylation plays an important role in contributing to the transcriptional regulation of various cells. Sufficient studies have shown the crucial roles of N6-methyladenosine (m6A)- and m6A-associated- regulators in embryogenesis during implantation. They are also essential in regulating innate and adaptive immune cells and the immune response and shaping the local and systemic immune microenvironment. However, the function of m6A modifications at the maternal-fetal interface still lacks wide research. This review highlights the critical functions of m6A in early embryonic development, summarizes the reported research on m6A in regulating immune cells and tumor immune microenvironment, and identifies the potential value of m6A modifications in shaping trophoblasts, decidual immune cells, and the microenvironment at the maternal-fetal interface. The m6A modifications are more likely to contribute to embryogenesis, placentation and shape the immune microenvironment at the maternal-fetal interface. Uncovering these crucial regulatory mechanisms could provide novel therapeutic targets for RNA methylation in early pregnancy.

## Introduction

Most mammalian genomes undergo RNA transcription, and many RNA transcripts can never be translated into proteins (1), which may lead to functional defects. RNA is not only an essential intermediate in the flux from DNA to proteins but also a regulatory molecule for fundamental cellular processes, the dysfunction of which contributes to important pathological processes (2). The coding and noncoding transcriptomes are widely and dynamically regulated by chemical modification, which adds new modifications of complexity and functionality to the emerging roles of RNAs in physiological and pathological conditions (3). Covalent modifications sense the changing environment directly and rapidly without changing the DNA and RNA sequences (4). In contrast to the epigenetic modifications on DNA and histones that work at the transcriptional level, RNA methylation has a notable effect on gene regulation at the posttranscriptional level (5). RNA modifications affect transcripts by altering the charge, base-pair potential, secondary structure and RNA–protein interactions, which, in turn, regulates gene expression *via* RNA processing, localization, translation and degradation (3). N6-methyladenosine (m6A) is the most abundant internal mRNA modification. Identifying of the proteins that mediate m6A modifications has elucidated the roles of mRNA modifications in nearly every aspect of the mRNA life cycle, as well as various cellular, developmental, and disease processes (3). In mammals, approximately 0.1%- 0.6% of adenines undergo m6A modification, with an average of 3-5 methylated sites in each mRNA. Remarkably, m6A modifications can be deposited onto transcripts in tissue- and cell-type-specific- manners (6). The m6A modifications commonly occur in yeast, plants, flies, bacteria, humans and other mammals, which implicates its multiple functions in RNA, including precursor mRNA (pre-mRNA) splicing, mRNA translation, stability, structure, export and decay, implying an association with several cellular processes, such as cell differentiation and reprogramming, further contributing to various human diseases (reviewed in (7)). For m6A detection, methods, including analytical chemistry, high-throughput sequencing, m6A-CLIP and miCLIP, have emerged to determine the specific methylation sites and the modification fractions at these sites to promote biological studies of RNA modifications (3, 8–11), which provided the available ways to study the roles of m6A modification proteins in physical and pathological processes.

Similar to DNA methylation, the deposition and removal of mRNA methylation also depend on a multiunit methyltransferase complex that was initially documented in 1994 (7). mRNA methylation is governed by three types of proteins, namely, methyltransferases as “writers”, demethylases as “erasers”, and specific m6A-binding proteins (YTHDF1-3) as “readers” (12). The deposition of m6A methylation is catalyzed by multicomponent methyltransferases, mainly methyltransferase-like 3 (METTL3), METTL14, Wilms’ tumor 1-associated protein (WTAP), RNA-binding motif proteins 15 (RBM15s), virlike m6A methyltransferase associated (VIRMA/KIAA1429), zinc finger CCCH-Type containing 13 (ZC3H13) and METTL16 (13–19). METTL3 is the most important component of the m6A methyltransferase complex (MTC) and highly conserved in eukaryotes from yeast to humans (20); METTL3 forms a stable heterodimer core complex regulated by WTAP and catalyzes the transfer of meth1 groups (13, 21). Similar to WTAP, RBM15s have no catalytic function but bind to METTL3 and WTAP to guide these two proteins to specific RNA sites for m6A modification. KIAA1429-mediated m6A methylation of mRNAs takes place near the 3’-UTR and the stop codon (22). m6A modification occurs when METTL3 and METTL14 are recruited into the nucleus (14). Only METTL3 has methyltransferase activity in the MTC. METTL16 encodes S-adenosylmethionine (SAM) synthase and is expressed in most cells. However, METTL16-mediated m6A sites were located in introns or intron–exon boundaries, which is different from the common m6A sites in UTRs (19). The m6A demethylases as “erasers” make the m6A methylation dynamic and reversible, which could be passively removed from the transcriptome *via* degradation of the modified RNA or active demethylation by the m6A demethylases fat mass and obesity-associated (FTO) or a-ketoglutarate-dependent dioxygenase alkB homolog 5 (ALKBH5), both belonging to the AlkB family of dioxygenases known to demethylate N-methylated nucleic acids (23, 24). FTO was the first demethylase discovered in 2011 (23). The ALKBH5 catalytic domain can demethylate m6A-containing single-stranded RNA (ssRNA) and single-stranded DNA (ssDNA) (25).

**Figure 1:**
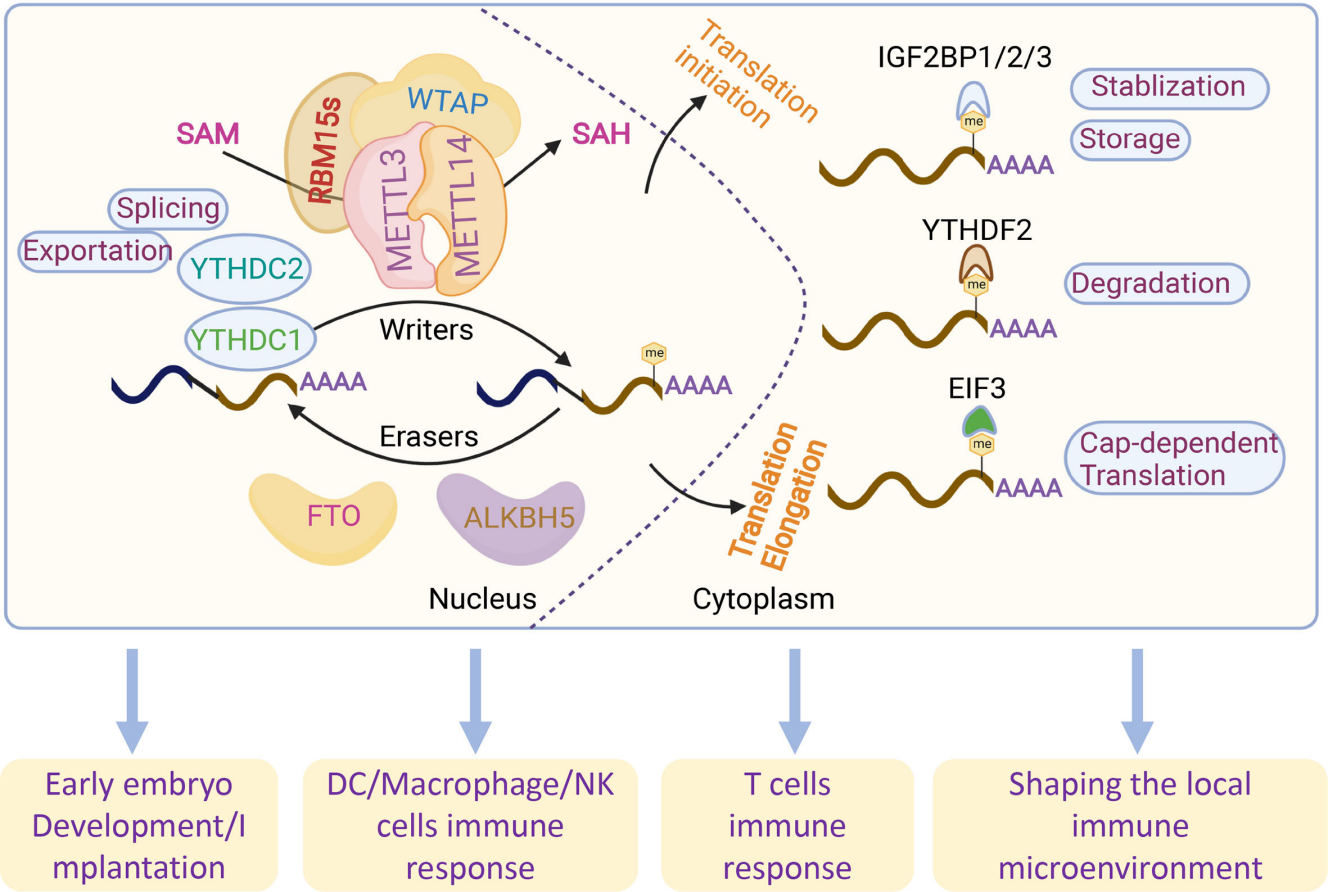
Overview of the reversible m6A RNA modifications and related functions. m6A modifications are regulated by m6A writers (METTL3, METTL14, RBM15s and WATP) and readers (FTO and ALKBH5). m6A methylation is recognized by different readers (YTHDC1-2, YTHDF1-3 and EIF3) by regulating RNA splicing, exportation, translation, degradation, stabilization and storage.

The discovery of reader proteins has made great progress in elucidating the impact of m6A methylation in mammalian cells. Different ‘readers’ with different cellular localizations influence almost all aspects of RNA metabolism, such as YT521-B homology domain family proteins (YTHDFs), insulin-like growth factor 2 mRNA-binding proteins (IGF2BPs), eukaryotic translation initiation factor 3 (EIF3) and heterogeneous nuclear ribonucleoproteins (HNRNPs) (26–29). Proteins with YTH domains located in the cytoplasm (YTHDF1, YTHDF2 and YTHDF3) and nuclei (YTHDC1 and YTHDC2) directly recognize m6A marks (27, 30), which promote the degradation and translation of m6A-modified RNA. In contrast to YTHDFs, IGF2BPs promote the stability and storage of their target mRNAs in a m6A-dependent manner under normal stress conditions and therefore affect gene expression output (28). EIF3 directly binds a single 5’ UTR m6A and recruits the 43S complex to initiate translation in a cap-dependent manner (26). Numerous studies have shown that m6A modifications play essential roles in multiple biological and pathological processes: hematopoietic development, central nervous and system development, the adaptive and innate immune system, carcinogenesis and the tumor microenvironment, as well as gametogenesis and early embryo development, the dysfunction of which generally results in various diseases by abnormal m6A modifications of the target genes (31) (**Figure 1**). Currently, with the development of assisted reproductive technology (ART), which helps to exclude abnormalities in the embryo, there remains the occurrence of recurrent pregnancy loss (RPL) in child-bearing women (32). Embryo implantation into the endometrium successfully relies on strict and coordinated regulation of trophoblasts derived from the fetus and decidual stromal cells and immune cells derived from the maternal sides (33). Crosstalk could be regulated by molecules associated with implantation, including hormones, signaling molecules, transcription factors and cytokines (34). However, the occurrence of RPL remains high, and more efforts should be made to discover the mystery of embryo implantation. Emerging studies emphasize the essential roles of m6A modifications in embryo implantation, while their roles in trophoblasts and immune tolerance at the maternal-fetal interface are worthy of further investigation.

Successful blastocyst implantation was first established by normal embryo development after fertilization. Its further development relies on the dynamically coordinated balance between the fetal-derived invading trophoblasts and the receptive maternal decidua (35). Once implantation is initiated, the trophoblasts, originating from the outer layer of the blastocyst, will differentiate into invasive extravillous trophoblasts (EVTs) to attach and invade the maternal decidua and therefore promote placentation (36), making the first contact with the maternal immune system. The placenta not only mediates the hormonal, nutritional and oxygen support to the fetus but also plays an essential immunoregulatory role at the maternal-fetal interface (37). Blastocysts that take paternal antigens as semiallogeneic to the maternal side can be recognized and accepted by the immunosuppressive maternal immune system (38). After normal implantation, the implantation sites are infiltrated with diverse immune cells, which are mainly characterized by an anti-inflammatory Th2-type immune microenvironment (39). Previously, embryo quality was shown to contribute to adverse pregnancy complications. ART tools make the selection of high-quality embryos available; however, implantation rates and successful pregnancy with ART are still relatively low. This indicated that uterine receptivity might also play a crucial role in the establishment of normal pregnancy (40). Diverse mechanisms, including cytokine/chemokine and hormonal signaling as well as DNA modifications, contribute to the pathology of pregnancy complications (40), which is attributed to the abnormal gene expression of cytokines/chemokines and abnormal signaling in a specific time and space during placentation and further fetal development.

Early mammalian embryos are capable of strong pluripotent stemness, which could be reprogrammed by epigenetic modifications. m6A modification is highly conserved from yeast to mammals and can regulate gene expression output, determine stem cell fate and cell differentiation, and further shape the local microenvironment. Recent studies have shown that m6A modifications are associated with animal reproductive processes, including gametogenesis, maternal-zygote transition (MZT) and early embryonic development (41–45). In addition, m6A modifications also play an essential roles in fine-tuning the immune response, including innate and adaptive immune responses and immune system development (8). Here, we reviewed the potential roles and novel insights of m6A methylation in embryo development and immune tolerance during early pregnancy during maternal-fetal tolerance.

## m6A functions in preimplantation embryogenesis

After fertilization, the newly generated zygote sustains a transcriptionally quiescent state and initiates early maternally programmed embryogenesis, following zygote genome activation (ZGA) with a clearance of maternal stores (RNA and DNA), which is termed the maternal-to-zygote transition (MZT) (46). The most important biological process, early embryonic development, is generally determined by a programmed transition into a totipotent and pluripotent embryonic state, followed by cell fate decisions and lineage-specific differentiation (47). Early embryogenesis relies on maternally inherited mRNA. Recently, emerging studies have reported that the epitranscriptomic mark m6A and its cofactors play critical roles in ensuring gene expression in an appropriate time and space in both preimplantation and postimplantation embryonic development (46, 47). In the murine preimplantation embryo, germinal vesicle (GV) oocyte-specific knockdown (KD) of *Mettl3* inhibited oocyte maturation and the MZT by disrupting maternal mRNA degradation (41). Additionally, *Mettl3* is mainly located in the intracisternal A particle (IAP)-type family of endogenous retroviruses. *Mettl3* knockout (KO) in mice blocked the integrity of multiple heterochromatin marks on METTL3-targeted IAPs. Mechanistically, the RNA transcripts in METTL3-bound IAPs are related to m6A-methylated chromatin, which is regulated by the m6A reader YTHDC1. This interaction, in turn, promotes the association of METTL3 with chromatin. Furthermore, METTL3 also interacts with the H3K9me3 methyltransferase SETDB1 and its cofactor TRIM28 (48). These results suggest an important role of METTL3-targeted IAP integrity in mouse embryonic stem cells. Downregulation of the m6A reader *HnRNPA2/B1*, which is regulated by *Mettl3*, blocked mouse embryonic development from the 4-cell stage by altering global gene expression involving the transcription, translation, cell cycle, embryonic stem cell differentiation, and RNA methylation pathways in *HnRNPA2/B1* KD blastocysts. Similar results were found in *Mettl3* KD blastocysts, which also showed that HnRNPA2/B1 is regulated in a Mettl3-dependent manner (49). In Mettl14 arginine 255 (R255me) mutant mice, embryonic stem cells (mESCs) led to decreased global mRNA m6A levels and preferentially affected endoderm differentiation in mESCs. Mettl14 R255me markedly enhances the interaction of Mettl3/Mettl14 with WTAP and binds to the substrate RNA. Moreover, protein arginine N-methyltransferase 1 (PRMT1) regulates Mettl14 at R255, which highlights the communication between protein and RNA methylation in regulating gene expression (50). Knockdown of Mettl3 and Mettl14 in mESCs led to similar phenotypes, with a lack of m6A RNA methylation and loss of self-renewal capability (27). WTAP, as part of the MTC, is essential for the blastocyst rate and global m6A levels of porcine early embryonic development, indicating the indispensable role of WTAP in porcine embryo development (51). An mRNA interactome capture study in zebrafish embryos identified the dramatic translocation of Hnrnpa1 accompanied by the movement from cytoplasmic to nuclear RNA targets and other pre-mRNA splicing factors to the nucleus in a transcription-dependent manner, indicating that Hnrnpa1 RNA-binding activities regulated RNA metabolism during early embryo development in a spatial and temporal manner (52). Accurately, one-third of zebrafish maternal mRNA is m6A modified, and m6A-binding protein promotes the clearance of maternal mRNAs, the removal of which slows down the decay of m6A-modified maternal mRNA and impairs ZGA, therefore blocking the initiation of the timely MZT and cell cycle and contributing to the overall delay of larval life (43). Additionally, the m6A reader protein YTHDF1-3, as a maternal mRNA-binding partner, was highly expressed in the zebrafish MZT process (3), suggesting that YTHDF protein-mediated m6A modification may regulate the MZT process. m6A-methylated maternal mRNA degradation impedes YTHDF2-deficient zebrafish embryos and therefore delays the timely MZT and leads to developmental interruption during the larval period (43). In addition, it was also reported that the m6A reader YTHDF2 is essential for oocyte maturation and embryo development (43, 53). The oocyte-specific deletion of YTHDF2 in mice also impeded the degradation of maternal mRNAs, thereby delaying the ZGA process. These results suggest that YTHDF2 plays important roles in the transcriptome transition by mediating m6A-dependent mRNA degradation. Similar to YTHDF2, the oocyte-specific deletion of VIRMA contributes to female-specific infertility in mice, which inhibits oocyte maturation by regulating pre-mRNA alternative splicing (54). In human ESCs, the ALKBH5 catalytic domain is fused to targeted RNA m6A erasure (TRME) and therefore demethylates the target m6A sites and increases mRNA stability with limited off-target effects (55). However, the role of VIRMA in early embryonic development remains unknown and requires further investigation.

**Figure 2:**
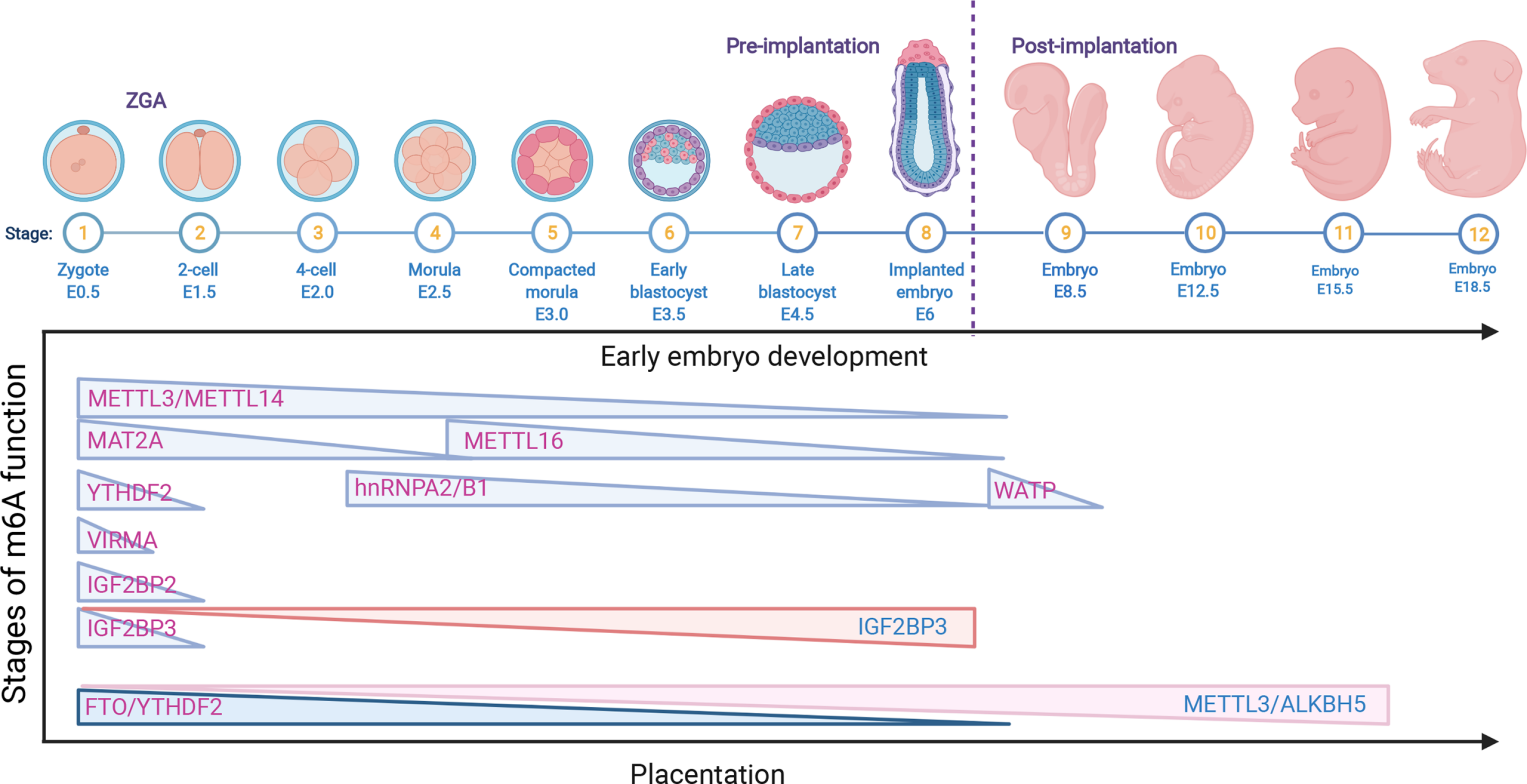
The role of m6A RNA modifications in early embryo development and placentation. The downregulation of the m6A RNA modifications METTL3, METTL14, METTL16, MAT2A, VIRMA, WATP, YTHDF2, hnRNPA2/B1 and IGF2BP2/3 is related to early embryo development; however, the upregulation of IGF2BP3 also causes a delay in late development. In addition, the decrease in FTO and YTHDF2 is associated with early placental dysfunction, while the overexpression of METTL3/ALKBH5 is related to late abnormal placentation.

In contrast to YTHDF2 that promotes mRNA decay, IGF2BPs could work as a new class of cytoplasmic m6A readers that regulate the stability and storage of mRNAs (28). Downregulation of *Igf2bp1* in zebrafish parthenogenetic activation (PA) embryos decreased the cleavage and blastula rates, which induced cell apoptosis and could be rescued by augmenting the miR-670 inhibitor (56). Maternal deletion of *Igf2bp2* (also called *IMP2*) results in murine early embryo development arrest at the 2-cell stage *in vitro* by decreasing the expression of *Ccar1* and *Rps14*, both of which are essential for early embryonic developmental competence (57). However, the role of IGF2BP2 in regulating mRNA stability and degradation in ZGA as a m6A reader still needs to be clarified. Deletion of maternal *Igf2bp3* degraded maternal mRNAs prior to MZT and resulted in severe developmental defects of abnormal cytoskeleton organization and cell division and destabilized the Igf2bp3-bound mRNAs. Interestingly, *Igf2bp3* overexpression in wild-type embryos also causes a developmental delay. These results indicate the important functions of Igf2bp3 in regulating early zebrafish embryogenesis by binding and stabilizing maternal mRNAs (58). The above findings suggested that the function of IGF2BP3 is different from that of YTHDF2, but both are indispensable for early embryogenesis in various species (**Figure 2**).

## m6A functions in postimplantation embryogenesis

After implantation, the blastocyst attaches to the uterus and initiates differentiation and development. The ectoderm of the blastocyst is the trophoblast stem cells, which differentiate into multiple trophoblast subsets and therefore promote placentation. The inner cell mass transitions from naïve state pluripotency to primed state pluripotency and is required for organogenesis and individual formation (47). A number of studies have reported that m6A determines the fate of embryonic stem cells (ESCs), losing m6A modifications causes pluripotent stem cells to display a state of hyperpluripotency and cannot differentiate into lineages, thus contributing to embryonic lethality (55, 59). Mechanistically, METTL3^-/-^ ESCs exhibited poor differentiation potential that prevented KO teratomas from forming the three germ layers. The abnormal expression of NANOG from E5.5 to E7.5 in epiblasts led to embryonic lethality. *Mettl3* and *Mettl14* KO mice both exhibited embryonic lethality at E6.5 (59, 60). These findings indicate the important role of MELLT3/METTL14 in early embryogenesis. Another m6A writer, METTL16, regulates human MAT2A, and increasing METTL16 binding to the methionine adenosyltransferase 2A (MAT2A) 3’UTR could promote efficient splicing in a hairpin (hp1) m6A-dependent manner (19). However, the m6A modifications on MAT2A were recognized by YTHDC1 for mRNA degradation (61). Although normal morphology and genotyping ratios were observed in E2.5 and E3.5 blastocysts from WT and *Mettl16* KO mice, only 1.9% *Mettl16* KO embryos at E6.5 could be found in the *Mettl16* ^-/-^ mouse model, indicating that METTL16 deletion led to embryo lethality around implantation (42). Interestingly, the most decreased gene in E2.5 KO embryos was *Mat2a*, suggesting that *Mettl16* and *Mat2a* are essential for early embryonic development. Apart from METTLs, another m6A writer, WATP-deficient ESCs, failed to differentiate into endoderm and mesoderm. In addition, deficiency of WATP in embryos results in abnormal egg cylinders at the gastrulation stage and causes embryonic lethality at E10.5 in mice (62). However, the relevant mechanisms need further exploration. YTHDC1, as the only nuclear reader, regulates the alternative polyadenylation (APA), AS and nuclear export of m6A-modified mRNAs in mouse oocytes. In addition, YTHDC1 is essential for early embryonic development (63). These results indicated that YTHDC1 is not only critical for gametogenesis but also important for the viability of early embryo development. Interestingly, no colonies were found in hnRNPA2/B1 mouse KO blastocysts. Nonetheless, knockdown of hnRNPA2/B1 impeded embryonic development after the 4-cell stage and blocked further development, and a similar phenotype was observed in Mettl3 KD embryos. Furthermore, Mettl3 KD blastocysts showed enhanced mislocalization of hnRNPA2/B1 and reduced m6A methylation, which suggested that hnRNPA2/B1 is important for early embryogenesis by Mettl3-dependent m6A RNA methylation (49). Deficiency of m6A methylation writers, easers and readers generally leads to embryo lethality postimplantation. However, these mutants focus on the embryo itself and not the placenta. The results of deciphering the developmental disorders programmed for placental phenotypes in embryonic lethal and subviable mouse knockout lines showed that 68% of KO lines mainly exhibited placental dysmorphologies (64). Early embryo lethality is closely associated with placental malformation, which strongly correlates with abnormal brain, heart and vascular development (64). The critical role of the placenta in pregnancy was determined by the trophoblast lineage. In preeclampsia (PE), METTL3 and m6A methylation were upregulated in the placental trophoblast (65). The maturation of miR-497/195-5P mediated by METTL3 impeded trophoblast migration and invasion by targeting WWP1 in PE patients (66). In addition, the RNA demethylase FTO and HLA-G were significantly decreased in the trophoblasts of spontaneous abortion (SA) patients, and the mRNA expression of *VEGFA, VEGFR* and *MMP2* bound to YTHDF2 also decreased in SA patients, which indicated that FTO in the chorionic villi promotes immune tolerance and angiogenesis at the maternal-fetal interface due to aberrant methylation and oxidative stress and therefore leads to the occurrence of SA (67). Downregulation of ALKBH5 demethylase KDM3B mediated activated leukocyte cell adhesion molecule (ALCAM) by increasing PPARG mRNA m6A modification and activating the Wnt/β-catenin pathway, in turn relieving PE progression (68). In addition, Xiaocui Li et al. reported that global mRNA m6A methylation was significantly decreased in villi from RPL patients without affecting ALKBH5 expression. Besides, ALKBH5 KD in villous explants enhanced trophoblast invasion by upregulating the half-life of cysteine-rich angiogenic inducer 61 (CYR61) mRNA (69) (**Figure 2**). Although some m6A modifications are reported to be associated with the differentiation and function of trophoblasts, more explorations are needed to elucidate the other m6A enzymes in the biological and immunoregulatory functions of trophoblasts and provide broad RNA epigenetic regulatory patterns in physical and pathological pregnancies.

## m6A functions in the innate immune response

**Figure 3:**
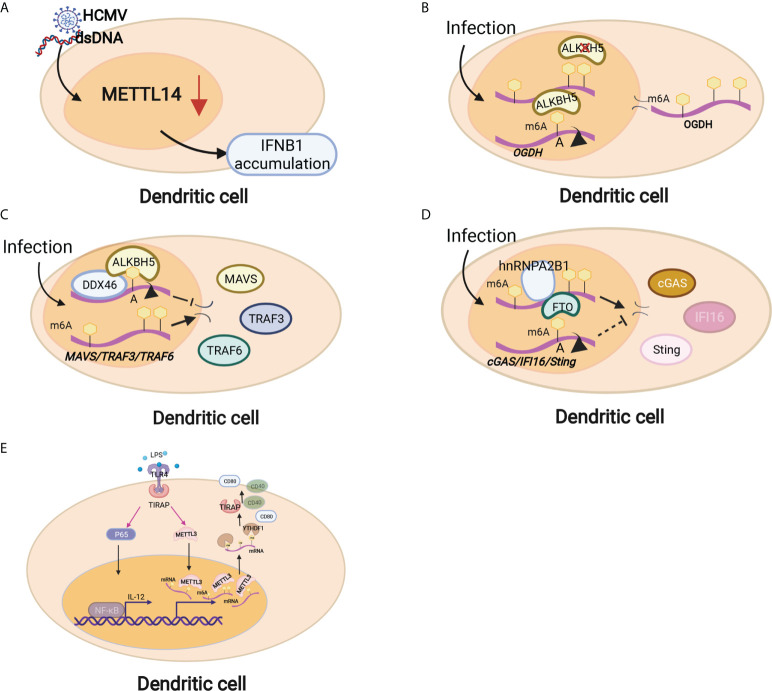
Dendritic cells are regulated by m6A modifications through different mechanisms. **(A)** METTL14 depletion inhibited viral reproduction and promoted dsDNA- or HCMV-induced IFNB1 mRNA accumulation. **(B)** Downregulation of ALBKH5 in DCs increased m6A modifications on OGDH mRNA and reduced its mRNA stability and protein expression, thereby inhibiting viral replication. **(C)** The RNA helicase DDX46 demethylates the antiviral proteins MAVS, TRAF3 and TRAF6 by recruiting ALBKH5. **(D)** hnRNPA2B1 recognizes viral DNA and facilitates m6A modification nucleocytoplasmic trafficking of CGAS, IFI16, and STING mRNAs by preventing FTO-mediated demethylation. **(E)** Mettl3-specific depletion in DCs results in delayed maturation in response to lipopolysaccharide (LPS) and impaired phenotypic and functional maturation of DCs. Mechanistically, the expression of the costimulatory molecules CD40 and CD80, the TLR4 signaling adaptor Tirap and the cytokine IL-12 decreased with a low capacity to stimulate T-cell responses.

Innate immunity provides the first line of defense against infections in a nonspecific manner. The innate immune cells at the maternal-fetal interface consist of macrophages (MΦs), natural killer (NK) cells and dendritic cells (DCs), which can sense invading pathogens and exogenous RNAs rapidly and thus respond in a timely manner to foreign pathogens (70). Innate immune cells comprise large populations of immune cells at the maternal-fetal interface (35). DC cells are the main antigen presentation cells (APCs) that can activate T cells and are equipped with the capacity to effectively take up, process and present antigens on the cell surface (71). Emerging studies have shown that m6A modification and m6A-associated proteins mediate innate immunity by regulating the recognition and responses to foreign pathogens, unmodified tRNAs, exogenous RNAs and aberrant endogenous RNAs. The recognition of foreign pathogens depends on several pattern-recognition receptors, such as plasma membrane receptors (Toll-like receptors, TLRs) and cytosolic sensors (RIG-I-like receptors, RIG-I and NLR proteins) (72). Kariko K et al. in 2005 reported that m6A modification decreased TLR3, TLR7, or TLR8 activation in monocyte-derived DCs (MDDCs), which was the first time that the regulatory effect on the process of RNA recognition was presented (73). Once RNA recognition occurs, the innate immune response immediately initiates and releases multiple cytokines, such as type I interferons (IFNs) and interferon-stimulated genes (ISGs) (74, 75). METTL14 depletion inhibited viral reproduction and promoted dsDNA- or HCMV-induced IFNB1 mRNA accumulation, while ALKBH5 depletion exhibited the opposite effect (75)(**Figure 3A**). The RNA helicase DDX46 demethylates the antiviral proteins MAVS, TRAF3 and TRAF6 by recruiting ALBKH5 (76) (**Figure 3C**). hnRNPA2B1 recognizes viral DNA and facilitates the m6A modification nucleocytoplasmic trafficking of CGAS, IFI16, and STING mRNAs by preventing FTO-mediated demethylation, thereby amplifying IFN production and enhancing the antiviral effect on HSV-1 infection (77) (**Figure 3D**). In addition, m6A modification also mediates the metabolic program to promote host immunity against viral infection. Downregulation of ALBKH5 increases the m6A modifications on the mRNA of a-ketoglutarate dehydrogenase (OGDH) and reduces its mRNA stability and protein expression, which inhibits viral replication (**Figure 3B**). These studies suggest that m6A modifications exert a contributory effect on antiviral responses by targeting antiviral-specific genes and proteins and reprogramming the metabolic state of the host. In addition, mounting evidence has shown that m6A methylation plays a critical role in DC activation and function. Mettl3-specific depletion in DCs results in delayed maturation in response to lipopolysaccharide (LPS) and impaired phenotypic and functional maturation of DCs. Mechanistically, the expression of the costimulatory molecules CD40 and CD80, the TLR4 signaling adaptor Tirap and the cytokine IL-12 decreased with a low capacity to stimulate T-cell responses (78) (**Figure 3E**). Loss of classical DCs enhanced the cross-presentation of tumor antigen and cross-priming of CD8+ T cells *in vivo*. Binding of YTHDF1 to transcripts of lysosomal proteases increases the translation of lysosomal cathepsins in DCs. Moreover, blockade of the PD-L1 checkpoint is enhanced in Ythdf1^−/−^ mice, implicating YTHDF1 as a therapeutic target in cancer immunotherapy (79). These findings indicate that m6A methylation and its related proteins play major roles in the maturation and activation of DCs and promote the initiation of the adaptive immune response through antigen cross-presentation.

**Figure 4:**
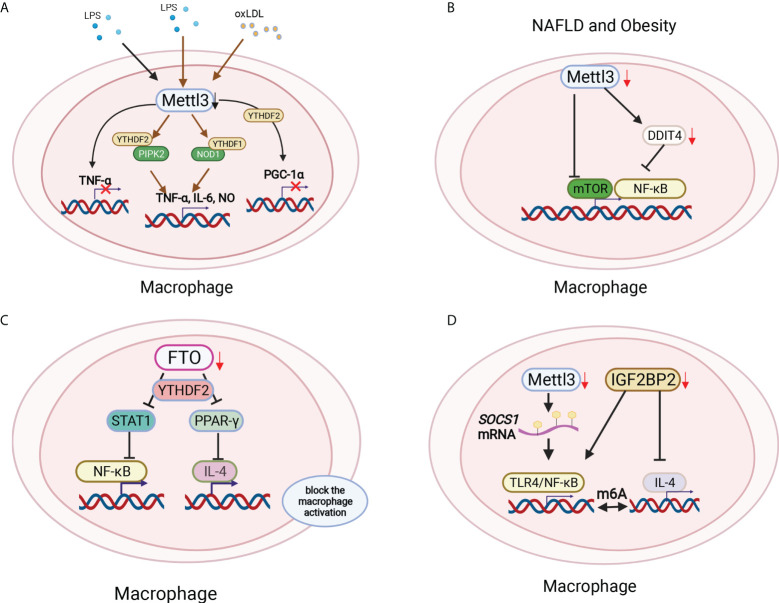
Macrophage polarization was modified by m6A methylation in different environments. **(A)** Mettl3 ablation macrophages produced little TNF-α with LPS stimulation. Mettl3 downregulation in macrophages significantly increased the proinflammatory cytokines TNF-α, IL-6 and NO by increasing NOD1 and RIPK2 *via* YTHDF1 and YTHDF2, respectively. Mettl3 and YTHDF2 cooperatively degraded PGC-1α mRNA in oxLDL-treated monocytes. **(B)** Myeloid lineage-restricted Mettl3 deletion protected mice from age-related and diet-induced development of innate immunity-driven nonalcoholic fatty liver disease (NAFLD) and obesity by decreasing mTOR expression and the NF-κB pathway by targeting DDIT4. **(C)** FTO deficiency inhibited both M1 and M2 polarization by suppressing the NF-κB signaling pathway and decreasing the stability of STAT and PPAR-γ *via* YTHDF2 involvement, therefore blocking macrophage activation. **(D)** The loss of Mettl14 decreased the demethylase Socs1 mRNA to activate TLR4/NF-κB signaling. In addition, IGF2BP2^-/-^ macrophages were refractory to IL-4-induced activation to regulate the switch of M1 to M2 subtypes in a m6A-dependent manner.

Macrophages serve as another main component of innate immune cells. RNA binding protein-focused CRISPR screening results showed that m6A writers were the top candidate genes in regulating LPS-activated macrophages. Mettl3 ablation macrophages produced little TNF-α with LPS stimulation (80) (**Figure 4A**). However, Mettl3 downregulation in macrophages significantly increased the proinflammatory cytokines TNF-α, IL-6 and NO. Mechanically, Mettl3 KO in macrophages promoted the expression and stability of NOD1 and RIPK2, which were mediated by YTHDF1 and YTHDF2, respectively (81) (**Figure 4A**). Additionally, Mettl3 and YTHDF2 cooperatively regulate PGC-1α mRNA degradation in oxidized low-density lipoprotein (ox-LDL)-induced monocytes (82)(**Figure 4A**). Mettl3 promotes the ox-LDL-induced inflammatory response in macrophages by modifying STAT1 mRNA, thereby polarizing macrophages to the M1 phenotype (83). The deletion of Mettl3 in myeloid cells promotes tumor growth and metastasis *in vivo*. Mechanistically, Mettl3-deficient mice showed increased M1/M2-like tumor-associated macrophage and regulatory T (Treg) cell infiltration in the local tumor microenvironment due to the impairment of YTHDF1-mediated SPRED2, which enhances the activation of nuclear factor κB (NF-κB) and STAT3 *via* the ERK pathway and consequently leads to tumor growth and metastasis. Furthermore, PD-1 checkpoint blockade was partially decreased in Mettl3-deficient mice, indicating the important role of Mettl3 in tumor immunotherapy (84). Additionally, myeloid lineage-restricted deletion of Mettl3 protects mice from age-related and diet-induced development of innate immunity-driven nonalcoholic fatty liver disease (NAFLD) and obesity. Mettl3 deficiency results in a notable increase in DNA damage inducible transcript 4 (DDIT4) mRNA. The decrease in mammalian target of rapamycin (mTOR) and NF-κB pathway activity in Mettl3-deficient macrophages could be restored by DDIT4 KD (85) (**Figure 4B**). These findings demonstrate the contribution of Mettl3-mediated m6A modification of DDIT4 to macrophage metabolic reprogramming in NAFLD and obesity. Lihui Dong et al. reported that macrophage-specific knockout of the m6A methyltransferase Mettl14 drives CD8+ T-cell differentiation with a dysfunctional trajectory, impairing CD8+ T cells to eliminate tumors (86). Silencing of the m6A eraser FTO markedly inhibited both M1 and M2 polarization by suppressing the NF-κB signaling pathway and decreasing the stability of STAT and PPAR-γ *via* YTHDF2 involvement, therefore blocking macrophage activation (87, 88) (**Figure 4C**). Mettl14 ablation in myeloid cells contributes to acute bacterial infection in mice by the continuous production of proinflammatory cytokines, which can be rescued by forced expression of Socs 1 in macrophages depleted of Mettl14 or YTHDF1. Loss of Mettl14 decreases demethylase expression, and Socs1 mRNA overactivates TLR4/NF-κB signaling. These findings highlight that m6A methylation-mediated SOCS1 expression is essential for the negative feedback control of macrophages on bacterial infection (89). YTHDF1 KD macrophages in rats improved the secretion of anti-inflammatory cytokines, highlighting the protective role of YTHDF1 KD macrophages in severe sepsis rats with ECMO (90). YTHDF2 KD in the mouse macrophage cell line Raw264.7 enhanced osteoclast formation and bone resorption (91). The m6A reader IGF2BP2-abated macrophages showed enhanced M1 polarization and promoted dextran sulfate sodium-induced colitis development. IGF2BP2^-/-^ macrophages are refractory to IL-4-induced activation by targeting tuberous sclerosis 1 to regulate the switch of M1 to M2 subtypes in a m6A-dependent manner, which indicates the key role of IGF2BP2 in the regulation of macrophages (92) (**Figure 4D**). These results indicated that the differentiation and function of macrophages could be regulated by m6A methylation.

**Figure 5:**
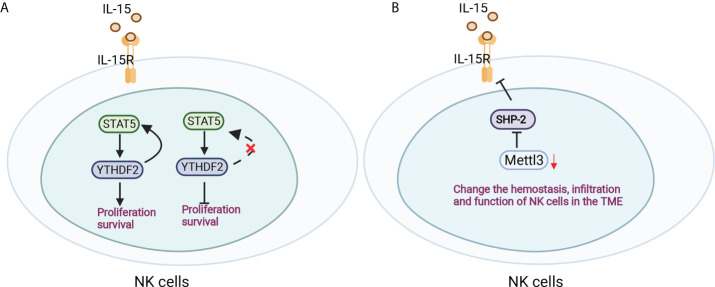
Antitumour immunity of NK cells was regulated by m6A modification. **(A)** YTHDF2 is essential for IL-15-mediated NK-cell survival and proliferation by the STAT5-YTHDF2 positive feedback loop. **(B)** Mettle3 ablation in NK cells changed the hemostasis, infiltration and function of NK cells by modifying SHP-2 mRNA in the tumor microenvironment.

NK cells are a large population of innate lymphocytes involved in antitumour and antiviral immunity. The m6A reader YTHDF2 is markedly increased in NK cells when activated by cytokines, tumors and virus infection, which mediates NK-cell antitumour and terminal maturation related to modulating NK-cell trafficking and regulating Eomes, respectively, losing which affects the antitumour and antiviral function of NK cells *in vivo*. Mechanistically, YTHDF2 promotes the effector function of NK cells and is essential for IL-15-mediated NK-cell survival and proliferation by the STAT5-YTHDF2 positive feedback loop, highlighting the novel biological role of YTHDF2 in NK cells in antitumour immunity (93) (**Figure 5A**). In addition, inactivation of Mettle3 in NK cells changed the hemostasis, infiltration and function of NK cells in the tumor microenvironment, leading to accelerated tumor growth and short lifespan in mice by modifying SHP-2 mRNA, which rendered NK cells hyporesponsive to IL-15 (94) (**Figure 5B**). However, the role of m6A modifications in the development and function of macrophages and NK cells remains limited and is worthy of more focus and investigation.

## m6A functions in the adaptive immune response

Adaptive immunity could be special in the clearance of specific pathogens, which are mainly mediated by the activation of antigen-specific T/B lymphocytes, and finally establish long-term immunological memory against the given antigen. Recently, an increasing number of studies have shown that m6A exerts an important effect on adaptive immunity and modulates the differentiation and function of different subsets of T cells (70). Val1, expressed in all immune cells, is used as Cre recombinase with the Val1 promoter in studying the immune response. However, METTL3 deficiency in Val1-Cre mice led to nonviable progeny, indicating the critical role of METTL3 in immune cells. While CD4, CD11C and Foxp3 were used to construct cell-specific transgenic mice (95), a large amount of m6A was still detectable (78, 96), which reflected incomplete METTL3 deletion.

The generation and maturation of CD4+ T cells in the thymus highly depend on the T-cell receptor (TCR) and multiple costimulatory signals. Initially, deficiency of METTL3 in CD4+ T cells did not affect the generation, maturation or capacity to respond to TCR stimulation *in vitro*, which indicated that the basic TCR signals and downstream signal transduction did not depend on m6A methylation *in vitro* (96). In CD4+ T-cell-specific *Mettl3* KO mice, the proportion of naïve cells was higher, while the proportion of activating CD4+ T cells was lower than that in WT mice, and *Mettl3* KO mice developed spontaneous colitis, indicating that m6A helped to keep naïve cells quiescent. A similar phenotype was observed in *Mettl14* KO mice (96).

**Figure 6:**
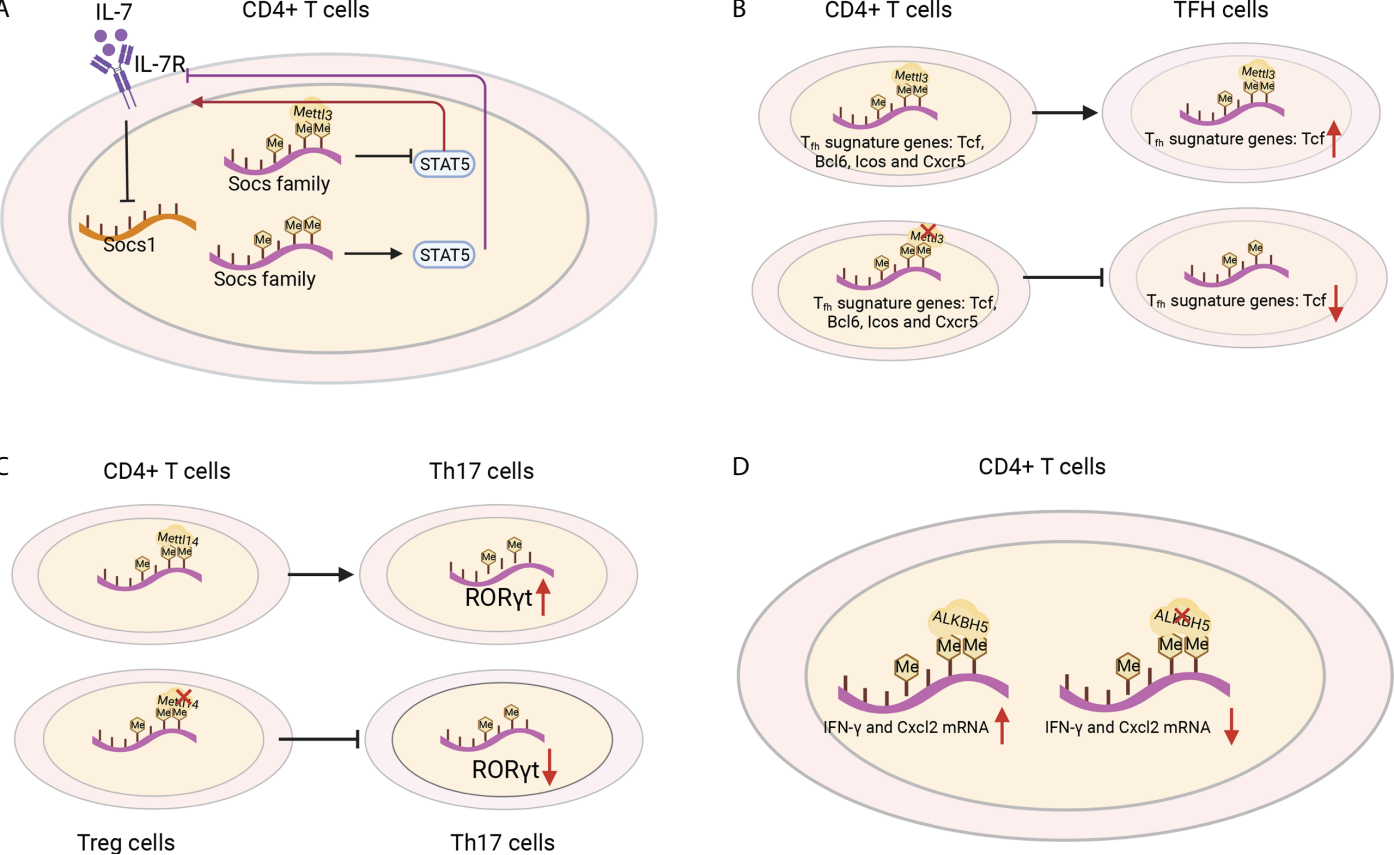
T-cell functions were shaped by m6A regulators. **(A)** IL-1R is a suppressor of SOCS1 targets. The Socs family prevents STAT5 activation and downstream signaling, which are marked by m6A and show slower mRNA degradation and higher protein expression in *Mettl3*-deficient T helper cells and blocked signal transduction through IL-7R. **(B)** Loss of METTL3 in CD4+ T cells leads to the degradation of the T_fh_ signature gene Tcf7 and inhibits T_fh_ cell differentiation. **(C)** Mettl14 ablation in Treg cells decreases RORγt expression and blocks the differentiation of naïve T cells into Th17 cells. **(D)** ALKBH5 deficiency in CD4+ T cells increases the m6A modification of IFN-γ and CXCL2 mRNA, thus decreasing their mRNA stability and protein expression in CD4+ T cells.

IL-7 is essential for homeostatic proliferation and long-term survival of naïve T cells (97). Likewise, the naïve T cells in *Mettl3* KO mice show a striking similarity to CD4+ T cells transferred to IL-7-deficient mice (98). The IL-7 receptor suppresses cytokine signaling of SOCS1 targets (98). Members of the SOCS family, including Socs1, Socs3 and Cish, bind the cytokine receptor and prevent STAT5 activation and downstream signaling (99) (**Figure 6A**). The SOCS genes were marked by m6A and showed slower mRNA degradation and higher protein expression in *Mettl3*-deficient T helper cells, which possibly impeded signal transduction through IL-7R (96). However, the role of m6A methylation in the response to cognate antigen recognition *in vitro* and pathogens *in vivo* cannot be excluded (8). T follicular helper (T_fh_) cells are specialized effector CD4+ T cells required for humoral immunity. The conditional deletion of METTL3 in CD4+ T cells inhibits TFH cell differentiation and the germinal center response in a cell-intrinsic manner. TFH signature genes, including Tcf7, Bcl6, Icos and Cxcr5, and these effects rely on intact methyltransferase activity. Loss of METTL3 results in accelerated decay of Tcf7 transcripts, emphasizing the role of Mettl3 in stabilizing Tcf7 transcription *via* m6A modification and T_fh_ cell differentiation (100) (**Figure 6B**). Whether CD8+ T cells and TCR signaling *in vivo* are regulated by m6A requires further investigation.

CD4+ regulatory T (Treg) cells represent differentiated CD4+ T cells that are transcribed by Foxp3, mediate immunosuppressive function and prevent the emergence of deleterious autoimmune diseases. Treg cells express high levels of IL-2R, which activates STAT5 and is essential for their immunosuppressive function (101–103). Although in Treg-specific *Mettl3* KO mice, the frequency of Treg cells was normal, both female and male mice developed severe autoimmune diseases and were infertile. Moreover, the mice died from 8-9 weeks and increased the mRNA levels of *SOCS* genes. These results suggest the important role of METTL3 in the immunosuppressive function of Treg cells (104). T-cell-specific Mettl14 deficiency induced spontaneous colitis in mice by increasing inflammatory cell infiltration, Th1/Th17 cytokines and the colonic weight-to-length ratio, which could be rescued by adoptive transfer of WT Treg cells. Mettl14-deficient Treg cells showed downregulated RORγt expression and blocked the differentiation of naïve T cells into Th17 cells (105)(**Figure 6C**). ALKBH5, not FTO, promotes naïve CD4+ T cells to induce adoptive transfer colitis. Additionally, T-cell-specific knockout ALKBH5 protects mice against EAE due to the increased m6A modification of interferon-γ and C-X-C motif chemokine ligand 2 (CXCL2) mRNA, thus decreasing their mRNA stability and protein expression in CD4+ T cells (**Figure 6D**). These changes resulted in an attenuated CD4+ T-cell response and diminished recruitment of neutrophils into the central nervous system, revealing the unexpected specific role of ALKBH5 in regulating the pathogenicity of CD4+ T cells in autoimmune disease (106). These studies highlighted some of the m6A modifiers in their therapeutic potential in antitumour and autoimmunity, and it would be interesting to decipher the regulatory networks in T cells and the functions of other RNA methylations in controlling T-cell differentiation, clonal expansion and their subsequent effector functions (107).

## m6A functions in shaping the local immune microenvironment

Emerging studies have shown that the local tumor microenvironment (TME) required for tumor growth and survival plays important roles in tumor development and progression. TME is complex and contains not only cancer cells and stromal cells but also macrophages and distant recruited cells, such as infiltrating immune cells, characterized by hypoxia, immune escape, metabolic dysregulation and chronic inflammation (108, 109). m6A has been widely investigated in regulating oncogenes or tumor suppressor genes in various cancers. Accumulating studies have recently reported a new role of m6A in the antitumour immune response. In addition to affecting classical immunotherapy, m6A also affects tumor-associated immune cell activation and infiltration and cytokine secretion in the tumor microenvironment, which play important roles in tumor initiation, progression, metastasis, and treatment response (110, 111). The tumor immune microenvironment (TIME) generally consists of the infiltration of multiple immunosuppressive cells, especially MDSCs and Treg cells, and is often absent of antitumour immune cells (111, 112).

**Figure 7:**
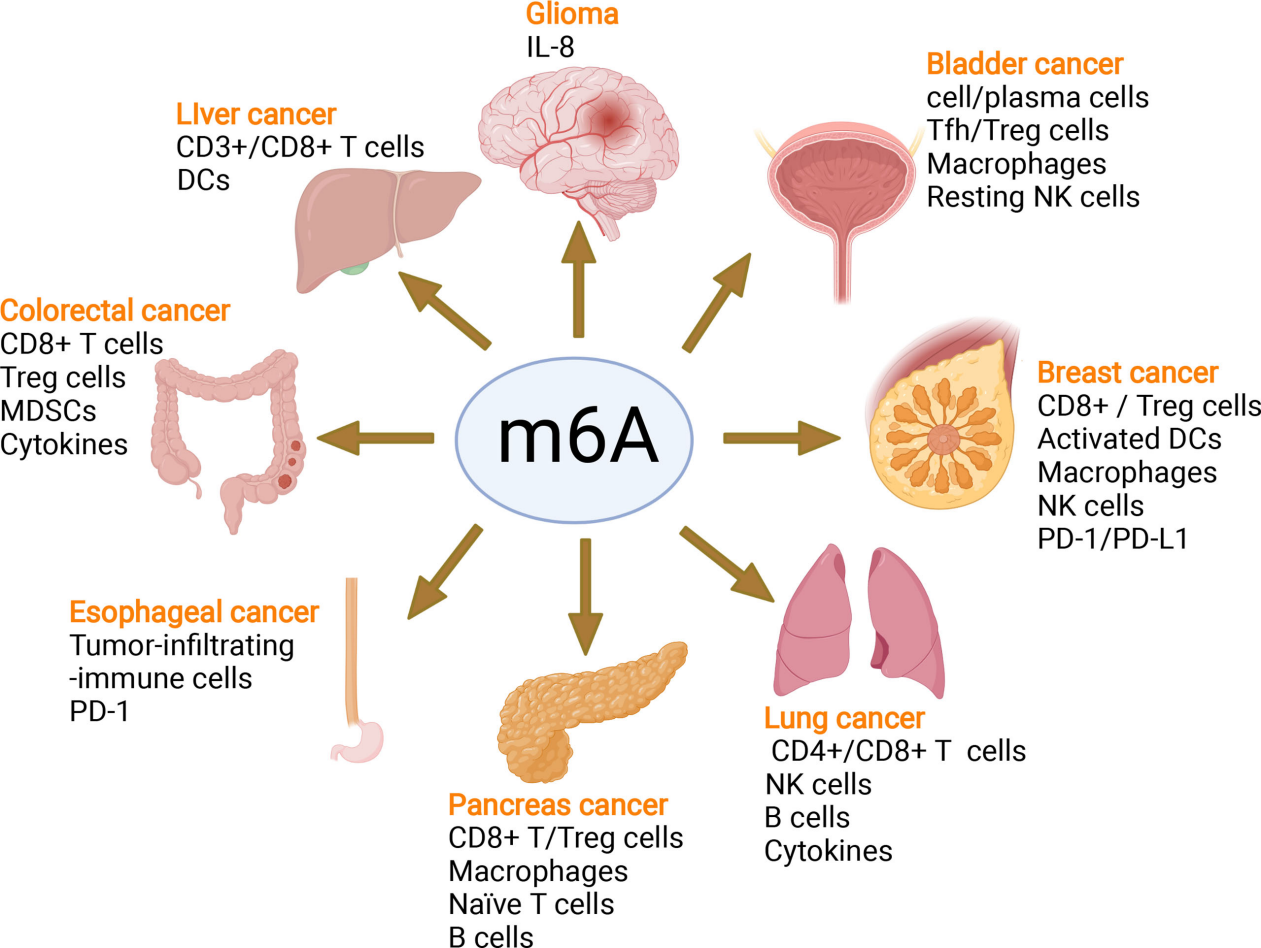
m6A modifies the tumor immune microenvironment and regulates tumor progression. m6A writers, erasers and readers regulate the tumor immune microenvironment in glioma, liver cancer, colorectal cancer, esophageal cancer, pancreatic cancer, lung cancer, breast cancer, and bladder cancer by controlling different immune cells and the PD-1/PD-L1 immune checkpoint mediating the efficacy of immune therapy.

**Figure 8:**
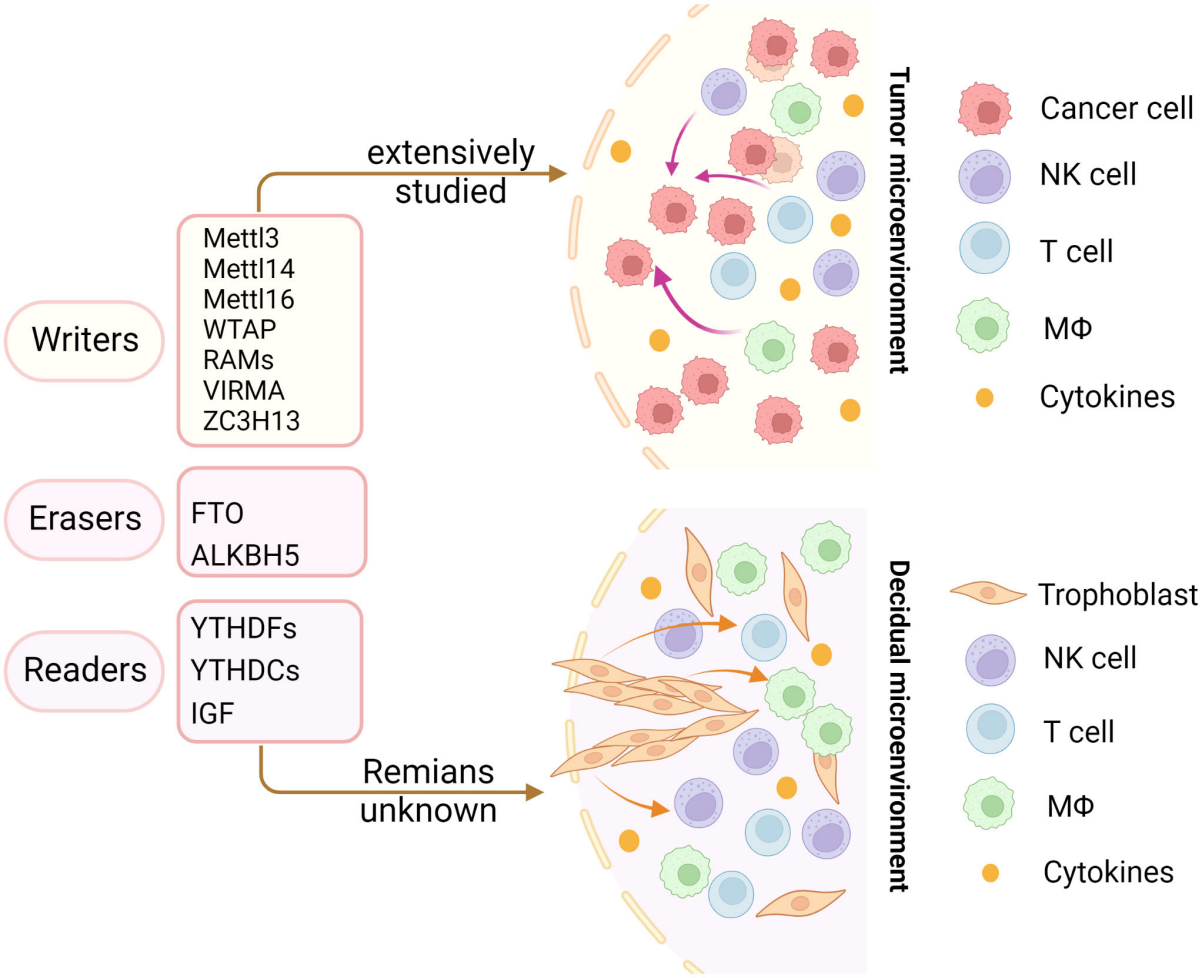
Insights into m6A modification in the tumor and decidual microenvironment. The m6A modifications in the tumor immune microenvironment have been extensively studied. However, the role of m6A modifications in decidual immune cells remains largely unknown. The same immune trajectory of decidual and tumor immune microenvironments encouraged us to study the mechanisms of m6A in decidual immune cells.

In recent years, emerging studies have deciphered the vital role of m6A modifications in the regulation of the local and systemic TIME, which mediate tumor progression and response to immunotherapy (113). The analysis of the m6A-related signatures from The Cancer Genome Atlas (TCGA), Genotype-Tissue Expression (GTEx) and the Gene Expression Omnibus (GEO) database revealed a significant relationship between the diverse m6A clusters and the TIME (114, 115). In glioma, m6A signatures were associated with classification, including prognosis, grade, isocitrate dehydrogenase (IDH) status and 1p19q status. Patients in the high-risk group showed enhanced stroma and immune scores and a higher abundance of immune infiltration (116–119). Silencing ALKBH5 in glioblastoma multiforme (GBM) notably suppresses hypoxia-induced tumor-associated macrophage (TAM) recruitment and immunosuppression in allograft tumors by regulating CXCL8/IL-8 secretion (120). Reduced METTL3 in hepatocellular carcinoma (HCC) results in increased infiltration of DCs in the TIME, which leads to the overall upregulation of major histocompatibility complex (MHC) molecules, costimulatory molecules, and adhesion molecules and is closely related to the prognoses of HCC (121). In addition, overexpressed YTHDF1 in HCC was associated with low CD3+ and CD8+ T-cell infiltration (122). ALKBH5 regulated PD-L1 mRNA in a YTHDF2-dependent manner on monocytes/macrophages and infiltration of myeloid-derived suppressor-like cells in the TIME of intrahepatic cholangiocarcinoma (ICC) (123). Alkbh5 in melanoma and colorectal cancers (CRC) regulates the metabolism/cytokines and infiltration of immunosuppressive Treg cells and MDSCs, therefore enhancing PD-1 immunotherapy and GVAX vaccination therapy (124, 125). In addition, Alkbh5 was related to the infiltration of monocytes in periodontitis, of which regulated m6A mediated the immune reaction of TNF-family-member receptors and cytokines, indicating the crucial roles of m6A in the diversity and complexity of the immune microenvironment of periodontitis (126). Additionally, Mettl3- or Mettl14-deficient tumors upregulated cytotoxic tumor-infiltrating CD8+ T cells and increased the production of IFN-γ, Cxcl9 and Cxcl10 in the TIME of CRC *in vivo*, thereby enhancing the response to anti-PD-1 treatment (127). METTL3, WTAP, IGF2BP3, YTHDF1, HNRNPA2B1 and HNRNPC were markedly increased in esophageal squamous cell carcinoma (ESCC) and positively related to the expression of PD-1, whose copy number dynamically affects the enrichment of tumor-infiltrating immune cells (128). Consensus clustering for 15 m6A regulators identified two molecular subtypes (clusters 1/2) in head and neck squamous cell carcinoma (HNSCC). Cluster 1 was enriched with G2 M checkpoint, mTORC1 signaling, and PI3K/AKT/mTOR signaling, while cluster 2 was associated with favorable prognosis, increased PD-L1, higher immune score and distinct immune cell infiltration (129). High-risk pancreatic adenocarcinoma (PAAD) contributed to the enhanced infiltration of M0 and M2 macrophages and decreased B cells, naïve T cells, CD8+ T cells and Treg cells (130–133). IGF2BPs, as functional downstream modulators of circNDUFB2, regulate the secretion of CXCL10, CXCL11, CCL5, and IFNβ in non-small cell lung cancer (NSCLC) (134, 135). In addition, m6A-related genes in peripheral blood leukocytes are noninvasive biomarkers for NCCLC patients (136). Nucleophosmin 1 (NPM1) is a chameleon protein that shuttles between the nucleus and cytoplasm. NPM1 is overexpressed in lung adenocarcinoma (LUAD) and effectively distinguishes LUAD from normal samples. The expression level of NPM1 in LUAD is markedly related to tumor stage and prognosis. Multiple database analysis showed that NPM1 is negatively related to B cells and NK cells. Moreover, NPM1 expression was significantly correlated with one m6A modifier-related gene YTHDF2 and five glycolysis-related genes (ENO1, HK2, LDHA, LDHB and SLC2A1) (137, 138). Four immune-related genes (IRGs), including CD274, CD8A, GZMA and PRF1, were screened and were consistent with the enrichment of CD8+ T cells and activated memory CD4+ T cells in the TIME of multiple cancers (132). In breast cancer, the three m6A clusters (writers, erasers and readers) are correlated with subsets of the infiltrating immune landscape, including activated CD8+ T cells, NK cells, activated DCs, macrophages and Treg cells. The low m6Ascore contributes to the increased mutation burden, immune activation and survival rates and is associated with an enhanced response to anti-PD-1/PD-L1 immunotherapy (139–141). In bladder cancer, 9 m6A-related lncRNAs were dramatically associated with overall survival outcomes of bladder cancer. The risk score of bladder cancer was correlated with the infiltration levels of multiple immune cells, including B cells, plasma cells, Tfh cells, Treg cells, resting NK cells, neutrophils, and M0, M1 and M2 macrophages, which indicated the important role of m6A-related lncRNAs in prognosis and shaping the tumor immune microenvironment (142, 143), which was also found in papillary thyroid carcinoma (PTC) (114) and HCC (144). The above studies indicated that m6A methylation and m6A-related modifications play essential roles in the differentiation and function of immune cells and secretion of cytokines, therefore shaping the TIME and further regulating the response to immunotherapy (**Figure 7**). The m6A modifications in the tumor immune microenvironment have been extensively studied (**Figure 8**). In addition, emerging studies have reported that m6A modifications can regulate the functions of multiple immune cells and cytokine secretion and shape the TIME, thus participating in the progression of cancer. Currently, RNA modification has become a new direction for studying embryo development and maternal-fetal immune tolerance. m6A modifications in the endometrium and ovary have been reported to be related to multiple gynecological diseases, including gynecological cancers, adenomyosis, endometriosis, polycystic ovary syndrome and premature ovarian failure, which generally contribute to RPL (145, 146). Researchers have proposed that receptivity at the maternal-fetal interface is more reminiscent of cancer immunology (35, 147). Trophoblasts and tumor cells share many similarities, including invasion, angiogenesis, and immunosuppressive environments, both of which are supported by an abetting microenvironment. However, the immunosuppressive environment in tumors severely influences antitumour therapy, which is different from the decidual immune environment (147). Emerging studies of m6A in tumors have shown potential value and strengthened the functions of m6A in the decidual local immune microenvironment. The crosstalk between trophoblasts and decidual immune cells, including decidual NK cells, macrophages and T cells, determines the local immune microenvironment at the maternal-fetal interface, the imbalance of which may lead to adverse pregnancy outcomes, such as RPL/RIF and preeclampsia (148). Although little is known about m6A methylation in trophoblast and decidual immune cells, knowledge of m6A modifications in shaping multiple immune cells and the TIME would inspire us to explore the potential roles of m6A functions in maternal-fetal immune tolerance by targeting trophoblasts and decidual NK cells, T cells and macrophages as well as cytokine secretion to shape the local immune microenvironment, thus affecting placentation and immune tolerance at the maternal-fetal interface, the imbalance of which may affect placentation and disrupt the receptive microenvironment and further lead to adverse pregnancy, including RPL and RIF. However, trophoblast cells are more precisely regulated than tumor cells, and the regulatory mechanisms of m6A in trophoblast and decidual immune cells remains unknown and requires further investigation (**Figure 8**).

## Conclusions and future perspectives

Numerous studies have elucidated the crucial roles of m6A- and m6A-associated regulators in embryogenesis during implantation. Additionally, other authors uncovered their essential roles in the differentiation and function of innate and adaptive immune cells as well as in shaping the local and systemic immune microenvironment. Indeed, m6A- and m6A-associated- regulators target cytokine secretion and multiple immune cells in the development and metastasis of various cancers by mediating the response to immunotherapy. Interestingly, the process of blastocyst implantation was similar to the metastasis of tumors; currently, the occurrence of RIF and RPL still disturbs child-bearing age women with unknown pathologies (35, 149). It is essential to determine the potential unknown factors causing RPL and RIF to provide clear targets for reproductive physicians. Although it is relatively straightforward to diagnose RPL, the progress of predicting and preventing RPL has been hampered by a lack of a standardized definition, uncertainties around the pathogenesis and highly variable clinical presentation. Moreover, the effectiveness of many medical interventions is controversial due to the available treatments targeting the putative risk factors for RPL (150). Therefore, it is urgent to explore the underlying pathologies that lead to RPL. Dysfunction of trophoblasts, stromal cells and decidual immune cells contributes to RPL and RIF. The m6A- and m6A-related- regulators participate in embryogenesis and shape the local microenvironment, which draws inspiration from studies in tumors and provides novel insight for investigating the potential pathologies causing RPL and RIF. Although some studies have reported the role of m6A- and m6A-related- regulators in the function of trophoblasts and are therefore correlated with pathological pregnancies, the relationship between m6A-and m6A-related- regulators and maternal decidual cells and the local immune microenvironment at the maternal-fetal interface is still unknown and needs further exploration, which would fill the gap in m6A in the shaping microenvironment at the maternal-fetal interface.

## Author contributions

HL: conceptualization, investigation, data curation, writing – original draft, visualization, funding acquisition. JZ: data curation, writing – review & editing. AL: conceptualization, writing – review & editing. All authors have read and approved the final manuscript.

## Funding

This work is supported by the Women and Children’s Hospital of Hubei Province (2021SFYY002).

## Acknowledgments

We thank Kahinho P Muyayalo and Zhibing Zhang for the language editing. The figures in this paper were created with BioRender.com.

## Conflict of interest

The authors declare that the research was conducted in the absence of any commercial or financial relationships that could be construed as a potential conflict of interest.

## Publisher’s note

All claims expressed in this article are solely those of the authors and do not necessarily represent those of their affiliated organizations, or those of the publisher, the editors and the reviewers. Any product that may be evaluated in this article, or claim that may be made by its manufacturer, is not guaranteed or endorsed by the publisher.
